# Homocysteine metabolism as the target for predictive medical approach, disease prevention, prognosis, and treatments tailored to the person

**DOI:** 10.1007/s13167-021-00263-0

**Published:** 2021-11-11

**Authors:** Lenka Koklesova, Alena Mazurakova, Marek Samec, Kamil Biringer, Samson Mathews Samuel, Dietrich Büsselberg, Peter Kubatka, Olga Golubnitschaja

**Affiliations:** 1 Clinic of Obstetrics and Gynecology, Jessenius Faculty of Medicine, Comenius University in Bratislava, 036 01 Martin, Slovakia; 2 Jessenius Faculty of Medicine in Martin, Biomedical Centre Martin, Comenius University in Bratislava, Mala Hora 4D, 036 01 Martin, Slovakia; 3 Department of Physiology and Biophysics, Weill Cornell Medicine in Qatar, Education City, Qatar Foundation, 24144 Doha, Qatar; 4 Department of Medical Biology, Jessenius Faculty of Medicine, Comenius University in Bratislava, 036 01 Martin, Slovakia; 5 Predictive, Preventive, Personalised (3P) Medicine, Department of Radiation Oncology, University Hospital Bonn, Rheinische Friedrich-Wilhelms-Universität Bonn, 53127 Bonn, Germany

**Keywords:** Predictive Preventive Personalized Medicine (PPPM/3PM), Homocysteine, Metabolism, Hyperhomocysteinemia (HHcy), Amino acids, Proteins, DNA methylation, Vitamin B6 and B12, Folate, Blood plasma, Molecular pathways, Remethylation, Transsulfuration, Diagnostic and treatment targets, Prognosis, Genetics, Epigenetics, Health risk assessment, Dietary habits, Nutrition, Systemic effects, Mitochondrial impairment, Cellular senescence, Cardiovascular risk, Endothelial dysfunction, Coronary artery disease, Ischemic stroke, Pregnancy complications, Oxidative stress, Inflammation, Impaired healing, Neurological disorders, Cancers, Eye disorder, COVID-19, Health policy, Primary, secondary, and tertiary care

## Abstract

Homocysteine (Hcy) metabolism is crucial for regulating methionine availability, protein homeostasis, and DNA-methylation presenting, therefore, key pathways in post-genomic and epigenetic regulation mechanisms. Consequently, impaired Hcy metabolism leading to elevated concentrations of Hcy in the blood plasma (hyperhomocysteinemia) is linked to the overproduction of free radicals, induced oxidative stress, mitochondrial impairments, systemic inflammation and increased risks of eye disorders, coronary artery diseases, atherosclerosis, myocardial infarction, ischemic stroke, thrombotic events, cancer development and progression, osteoporosis, neurodegenerative disorders, pregnancy complications, delayed healing processes, and poor COVID-19 outcomes, among others. This review focuses on the homocysteine metabolism impairments relevant for various pathological conditions. Innovative strategies in the framework of 3P medicine consider Hcy metabolic pathways as the specific target for in vitro diagnostics, predictive medical approaches, cost-effective preventive measures, and optimized treatments tailored to the individualized patient profiles in primary, secondary, and tertiary care.

## Physiologic Hcy levels and severity of deviations: what is the most optimal Hcy concentration in blood?

**Fig. 1:**
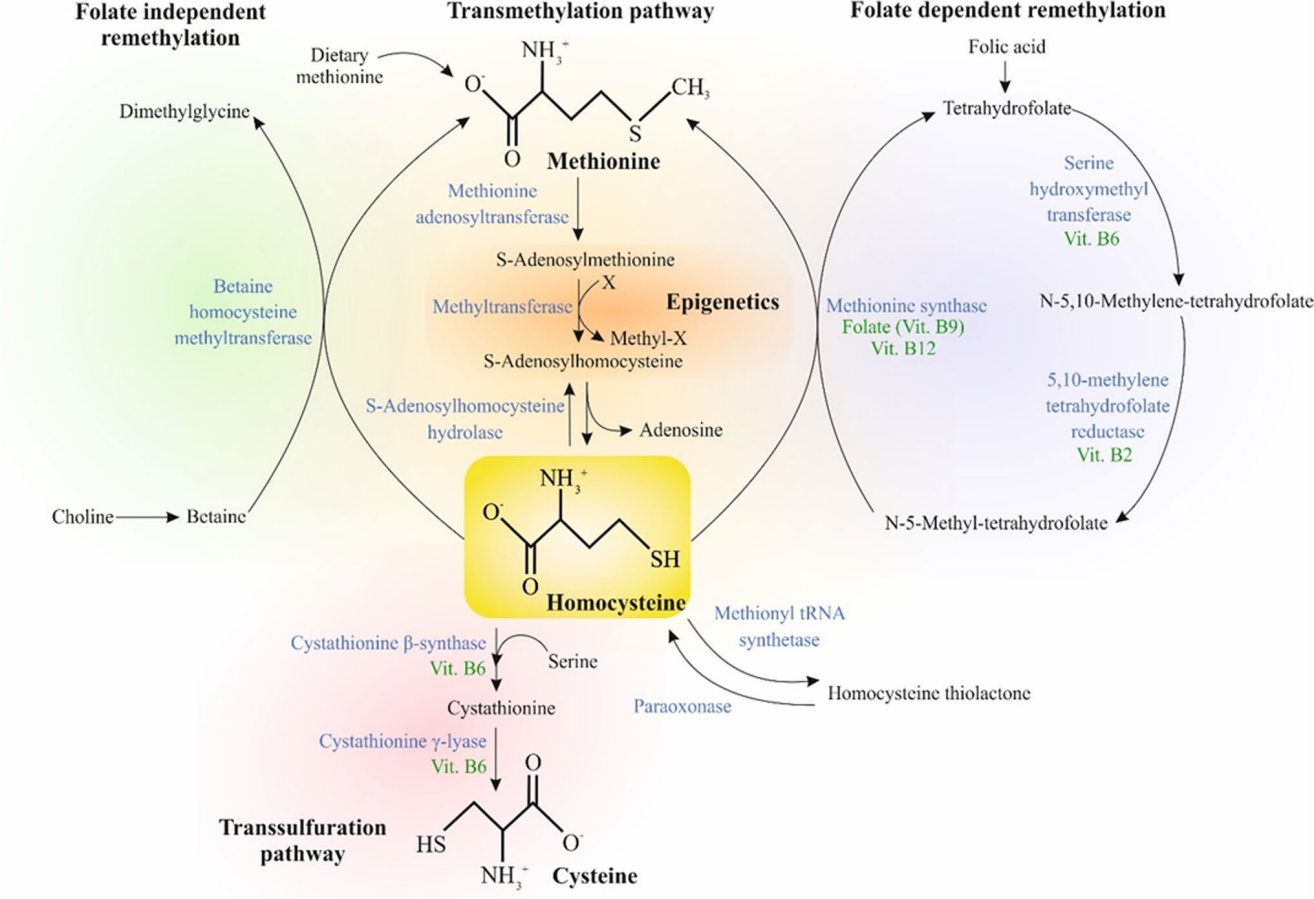
Hcy metabolism. Abbreviations: blue-colored words, enzymes; green-colored words, cofactors

Homocysteine (Hcy), a sulfhydryl-containing non-proteinogenic amino acid, is a metabolic intermediate produced by the demethylation of methionine (Met) in the body and is physiologically essential for processes such as cell cycle progression and maintenance of cellular homeostasis [1]. In turn, Hcy metabolism contributes to (1) the folate-dependent/independent remethylation to form Met and (2) the transsulfuration pathway (via cystathionine) to form cysteine. Both these pathways require vitamin-derived cofactors, including pyridoxine (vitamin B6), for transsulfuration pathway mediated synthesis of cysteine as well as folate (vitamin B9), cobalamin (vitamin B12), and riboflavin (vitamin B2) in the Met synthesis cycle (Fig. 1). These pathways are coordinated by S-adenosylmethionine (SAM), which have a specific role as an allosteric inhibitor for the methylenetetrahydrofolate reductase (MTHFR) reaction and acts as an activator of cystathionine β-synthase (CBS) [2]. In the presence of sufficient Met, Hcy produces cysteine through the enzyme cystathionine β-synthase [3]. However, in the event of Met deficiency, Hcy can be remethylated to salvage Met through the enzyme N5, N10-methylenetetrahydrofolate reductase [4]. Although Hcy is not directly involved in protein synthesis, its specific function in folate metabolism and choline catabolism is crucial for regulating Met availability and function [5].

Hcy is commonly found in blood plasma in four different forms: circulates as free thiol (1%), remains disulfide-bound to plasma proteins such as albumin (70–80%), and combines with other Hcy to form the dimer Hcy or combines with other thiols (20–30%) [6]. In healthy humans, the optimal total concentration of Hcy (tHcy) in plasma is in the range of 5.0 and 15.0 μmol/L (high-performance liquid chromatography method) or 5.0–12.0 μmol/L (immunoassay method). Fluctuations in Hcy levels are associated with various diseases, making Hcy to a useful marker of impaired amino acids and protein homeostasis [7, 8].

Elevated levels of Hcy in blood plasma (> 15 µmol/L) is a systemic medical condition known as hyperhomocysteinemia (HHcy) [9]. Furthermore, the range of Hcy between 16 and 30 μmol/L is classified as moderate, 31–100 μmol/L as intermediate, and above 100 μmol/L as severe HHcy [6]. Several risk factors such as aging, smoking, and oxidative stress contribute to HHcy severity [10, 11] contributing to severe pathologies, namely, neurodegenerative disorders (Alzheimer’s (AD) and Parkinson’s disease (PD), dementia, neuropsychiatric illness), thrombosis, cerebrovascular disease, osteoporosis-associated fractures, cardiovascular disease (CVD), and cancer [12–17]. Consequently, HHcy is associated with increased all-cause mortality [18].

Hypohomocysteinemia (< 6 μmol/L) occurs in 0.5–1% of the population [19]. Similarly to HHcy, also abnormally low Hcy concentration is considered a health risk factor. Despite a high prevalence of HHcy in patients receiving maintenance hemodialysis, a decreased blood plasma concentration of Hcy in this patient cohort correlates with increased hospitalization and mortality [20]. Further, low Hcy levels are associated with peripheral neuropathy (41% of patients with idiopathic peripheral neuropathy) [19]. In rare cases, low Hcy levels are associated with excessive conversion to cystathionine in the transsulfuration pathways leading to the impaired ability for de novo production of the anti-oxidant glutathione thus increasing the susceptibility to the oxidative stress overload [21]. Therefore, Met, N-acetylcysteine, and taurine supplementation is strongly recommended for patients with abnormally low levels of Hcy in blood plasma [21].

## HHcy-affected pathways and cascading pathologies

HHcy is often related to age and race physiological particularities as well as individual genetic, epigenetic, nutritional, and latrogenic (drugs) risk factors, among others [22]. At the same time, the leading cause of HHcy is related to an insufficient amount and/or dysfunction of enzymes and cofactors (water-soluble vitamins B2, B6, B9, and B12) associated with the metabolism of Hcy, especially in the elderly population [23, 24]. HHcy can be related to increased Hcy production by transmethylation, decreased Hcy removal by transsulfuration or remethylation, or a decrease in the Hcy excretion [25] as summarized in Fig. 1.

### HHCy is associated with disruptions in transmethylation pathway

Disturbances in the transmethylation pathway are related to HHcy characterized by an increased S-adenosylhomocysteine (SAH) and decreased SAM/SAH ratio, aberrant protein repair mechanisms requiring methyltransferases, and DNA hypomethylation [26]. SAH hydrolase deficiency caused by the missense mutation (R49H) in adenosylhomocysteinase (*AHCY*) in patients with liver disease and related increase in serum aminotransferases, SAH, SAM, and Met may lead to the early onset of hepatocellular carcinoma [27].

The transmethylation pathway is closely associated with epigenetic processes, including DNA methylation and histone modifications (acetylation, methylation, and N-homocysteinylation). However, these epigenetic changes also depend on several factors such as gender, diet, and/or gene mutations [28]. DNA methylation is connected to Hcy metabolism through the generation of SAM and SAH. In HHcy, the accumulation of SAH causes the decline of methylation capacity characterized by decreased SAM/SAH ratio [29]. A lack of essential one-carbon nutrients, including Met, folic acid, or choline, significantly reduces SAM and SAM/SAH ratio associated with decreased global DNA methylation. Despite the reversible changes of DNA methylation, a long-term administration (> 18 weeks) of a methyl-deficient diet causes irreversible DNA hypomethylations [30]. Further, excessive Hcy can be converted to a Hcy thiolactone that can react with the ε-amino group of a protein lysine residue (N*-*homocysteinylation) and contribute to the manifestations of HHcy [31]. Further, N-Homocysteinylation of both non-histone and histone residues (by increased Hcy thiolactone) represents a post-translational modification [32] that causes also alterations in gene expression [33].

### Folate-dependent and -independent remethylation

HHcy is associated with disruptions in the folate-dependent remethylation of Hcy to for Met. Folate, the water-soluble B9 essential vitamin, is a coenzyme in nucleic acid synthesis and Met regeneration [34]. Mild HHcy is usually caused by mild impairment of the methylation pathway and is associated with folate or B12 deficiencies or the thermolability of MTHFR [2]. Furthermore, a rare but severe form of HHcy is connected to genetic mutations of the enzymes implicated in Hcy metabolism resulting in MTHFR deficiencies or enzymes involved in methyl-B12 synthesis and Hcy methylation [2, 3]. HHcy caused by an altered expression of *MTHFR* is described as occurring in elderly patients with memory loss [35], cognitive impairments, AD, PD, epilepsy [36], and its C677T polymorphism is connected to a higher risk of various CVDs and associated morbidity and mortality [37]. Further, Hcy remethylation, primarily through a folate-dependent pathway, is markedly decreased in renal patients on hemodialysis; however, the transsulfuration pathway is not altered [38].

HHcy could also be connected to disruptions in folate independent remethylation of Hcy to form Met. Choline, a water-soluble vitamin-like nutrient, exerts heterogeneous functions in cells. One of its many functions is its specific role in Met regeneration. Choline can be oxidized to betaine that acts as an osmoregulator. Choline and betaine represent the essential sources of one-carbon units, especially during folate deficiency. Therefore, Hcy in the liver and kidney can be converted to Met by betaine-homocysteine methyltransferase (BHMT) [39, 40]. Dysfunction of BHMT leads to HHcy, which is associated with increased susceptibility to noise-induced hearing loss [41].

### HHcy-associated disruptions of the transsulfuration pathway

When extracellular cysteine is depleted, the production of cysteine through the transsulfuration pathway supports glutathione synthesis and protein translation [42]. On the other hand, the changes in transsulfuration pathway caused mainly by altered enzyme activity can be associated with HHcy and hyperhomocystinuria. A deficiency in CBS that converts Hcy into cystathionine increases Hcy levels. Also, Hcy concentration increases due to the widely diffused polymorphisms of several enzymes. Specifically, a T833C polymorphism in CBS is linked to mild HHcy in different ethnic groups [43]. Further, the 1364 T/T mutation of the cystathionine-γ-lyase gene also affects the enzyme cystathionine γ-lyase that is associated with the elevation of tHcy [35]. Both enzymes, CBS and cystathionine-γ-lyase, are responsible for hydrogen sulfide (H_2_S) generation through desulfuration reactions. The dysfunction of these enzymes can lead to HHcy and contribute to pathological oxidative stress, inflammation, cardiovascular and cerebral dysfunction, fatty liver disease, and ischemia–reperfusion injury [44].

## Diet-associated HHcy

Hcy plasma levels can markedly fluctuate among different populations due to their dietary habits [45]. Depending on the content of dietary Met, commonly found in poultry diet [46], and choline, approximately 50–80% of generated Hcy, is remethylated to Met [47]. In humans, the relation between Met intake and HHcy also depends on vitamin status (folate, vitamins B6 and B12) and the supply of other amino acids [48]. In vivo analysis revealed that a high-Met diet can induce HHcy and can affect epigenetic processes, mainly increased global methylation (5-mC) and DNA methyltransferase-1 (DNMT1) expression. Further, HHcy is associated with increased methylation of CBS promoter in bone marrow-derived endothelial progenitor cells [49].

HHcy is also related to the deficiencies in cofactors, like vitamin B12, B6, and folate, all are important for Hcy metabolism. The source of vitamin B12 is dairy and meat products [50]. Therefore, in vegetarians, dietary deficiency of vitamin B12 can cause HHcy, especially in the Indian subcontinent, Mexico, Central and South America, and some specific areas in Africa [51]. Regarding a plant-based diet in the Spanish population, a higher Hcy level is described in lacto-ovo vegetarians than vegans [52]. Further, folate is found in fresh food sources, including broccoli, brussels sprouts, or leafy green vegetables [53]. Therefore, tHcy levels and serum folate can fluctuate depending on the folate intake and genetic polymorphisms in MTHFR, such as C677T [54]. HHcy caused by inadequate ingestion of folate and vitamin B12 in diet can increase the risk of cervical artery dissection [55] or can promote oxidative stress in patients with type 2 diabetes [56]. Like most B vitamins, vitamin B6 is abundant in meat, fish, and poultry [57], and its deficiency leads to HHcy, which correlates with increased mortality from any cause [57].

For adults, the adequate intake for choline is 550 mg/day for men and 425 mg/day for women [58]. The dietary sources of choline and its derivatives phosphocholine, glycerophosphocholine, phosphatidylcholine, sphingomyelin, total choline, and betaine are summarized by the US Department of Agriculture (USDA). Whole eggs, organ meat, caviar, fish, and shiitake mushrooms represent the primary source of choline in diet [59]. Furthermore, spinach, cereals, grains, and grain-based products are primary dietary sources of betaine [60, 61]. Impaired folate independent remethylation by the deficiency in dietary choline and/or betaine can be related to HHcy. In rats with low-Met diet (standard soybean protein diet and low casein diet), the deprivation of choline induces HHcy, probably due to the inhibition of Hcy removal by both remethylation and cystathionine formation [62]. However, betaine or spinach can suppress HHcy induced by choline deficiency in vivo [61].

## Association of Hcy-axes with mitochondrial dysfunction

Mitochondria are essential for maintaining cellular homeostasis and function, primarily in oxidative phosphorylation (OXPHOS), and for regulation of ion homeostasis, redox potential, lipid metabolism, metabolite synthesis, cell differentiation, immune system, anti-apoptotic, and anti-aging mechanisms [63]. The disruption/disbalance of mentioned processes or accumulation of mutations in mitochondrial DNA (mtDNA) are related to mitochondrial dysfunction and aging-associated pathologies, including neurological disorders, CVDs, metabolic syndromes, and cancers [64]. In aging, progressive mitochondrial dysfunction occurs due to the loss of the thioretinaco ozonide oxygen ATP complex from mitochondrial membranes through the opening of the mitochondrial permeability transition pore [65]. Further, various studies describe the potential correlation between mitochondrial dysfunction and higher Hcy levels. In rats with acute myocardial ischemia–reperfusion injury, elevated plasma Hcy induced mitochondrial dysfunction and oxidative stress through increased cytochrome *c* release, stimulation of ROS production, and ERK1/2 signaling pathway that subsequently caused cardiac dysfunction [66]. Interestingly, HHcy is also implicated in elderly frailty and causes skeletal muscle weakness and fatigability. HHcy may cause mitochondrial dysfunction through reduced dystrophin levels along with a decrease in mitochondrial transcription factor A (mtTFA) and its regulator nuclear respiratory factor 1 (NRF-1) in rodent model [67]. To this end, elevated Hcy levels inhibit the enzymatic activity of mitochondrial complex I–III that is associated with higher cytochrome *c* release in rat ischemic brain as a model of cerebral infarction-related disease. In Hcy-treated animals, increased 8-hydroxy-2′-deoxyguanosine (8-OHdG) content and mitoStat3 protein phosphorylation were also observed. Finally, treatment with Hcy aggravated the damage of mitochondrial ultrastructure in the brain cortex and the dentate gyrus region of the hippocampus after focal cerebral ischemia [68]. Furthermore, prolonged Hcy treatment induced mitochondrial apoptosis of human umbilical vein endothelial cells through increased NADPH oxidase 4 (NOX4) expression and intracellular ROS production and decreased Bcl-2/Bax ratio and mitochondrial membrane potential (MMP), resulting in cytochrome *c* release and caspase-3 activation [69]. In PD rat model, Hcy also reduced activity of mitochondrial complex I and caused oxidative stress in the nigrostriatal pathway that were associated with increased production of hydroxyl radicals, reduced glutathione level, and enhanced activity of antioxidant enzymes such as superoxide dismutase and catalase [70].

Several aging-associated pathologies are characterized by altered mitochondrial functions. In many cases, the elevated Hcy levels aggravated mitochondrial dysfunction, resulting in poor prognosis. Therefore, in the treatment of mitochondrial impairment diseases, it is important to consider also Hcy levels in organism.

## Association of Hcy-axes with cellular senescence and aging

Cellular senescence and aging act as the risk factors that contribute to HHcy [10, 11]. Normal diploid fibroblast cells can divide approximately (45 to 50 times) in culture until the mitotic activity ceases. This phenomenon of cellular senescence is known as Hayflick limit. Cellular senescence is characterized by the shortening of telomeres and decreased activity of telomerase [65]. The efficiency of Hcy thiolactone metabolism declines during aging process that is related to decreased formation of SAM associated with the loss of thioretinaco ozonide. Thioretinaco ozonide can prevent carcinogenesis and atherogenesis; however, its loss from mitochondrial membranes underlying the aging process of cellular senescence [71].

Various studies revealed that Hcy can accelerate the cellular senescence through many mechanisms. In a study of Zhang et al. (2015), the exposure of cultured endothelial cells to Hcy led to cellular senescence through shortened telomeres via DNA hypomethylation of human telomerase reverse transcriptase (hTERT) and increased marker of cellular senescence acidic β-galactosidase. Further, Hcy upregulated the markers of cellular senescence, including p16, p21, and p53, in cultured endothelial cells; however, the administration of folic acid or SAM could reverse mentioned effect [72]. Furthermore, chronic exposure of endothelial cells to Hcy accelerated the rate of cellular senescence through the redox pathway suggesting that oxidative stress could increase the production of vascular cell senescence proven by increased expression of two surface molecules such as intracellular adhesion molecule-1 (ICAM-1) and plasminogen activator inhibitor-1 (PAI-1), factors implicated in the pathogenesis of atherosclerosis [73]. The exposure of cultured endothelial progenitor cells (EPC) to Hcy, precursors of mature endothelial cells, decreased proliferation and increased EPC senescence through diminished telomerase activity and Akt phosphorylation. However, the treatment with atorvastatin revealed the preventive effect against Hcy-induced senescence of EPC as a model of coronary heart disease [74].

The effects of aging on enzyme activity, connective tissues, lipid synthesis, auto-immune diseases, atherogenesis, and carcinogenesis are closely associated with changes in Hcy metabolism [71]. Elevated level of Hcy contributes to the acceleration of cellular senescence. It is important to evaluate the effects of various agents that could prevent or reverse the cellular senescence, thus inhibiting the progression of associated diseases.

## CVDs are associated with elevated plasma Hcy

CVD represents the leading cause of death in the world [75]. CVD includes several pathologies from which the coronary heart disease, cerebrovascular disease, or rheumatic heart disease have the highest incidence. More than 80% of CVD deaths are attributed to heart attacks and strokes, and approximately one-third of these deaths occur prematurely in people under 70 years of age [76]. Conventional risk factors of CVDs such as dyslipidemia, hypertension, smoking, or diabetes mellitus do not fully clarify all CVD events and deaths. Several studies summarized that elevated plasma Hcy denotes an independent risk factor for CVDs other than conventional risk factors and can be applied as a biomarker to predict CVD onset in individuals [77–79].

Mechanisms by which Hcy supposedly induces vascular injury and consequent CVDs include endothelial injury, DNA dysfunction, elevated oxidative stress, increased proliferation of smooth muscle cells, downregulation of glutathione peroxidase activity, and supporting the process of inflammation. These pathologic changes caused by Hcy are manifested by impaired flow-mediated vasodilation, mainly due to decreased nitric oxide (NO) production and bioavailability [80]. Unbalanced NO synthesis causes and potentiates oxidative stress and atherothrombogenesis. The damage of endothelial cells represents a crucial inducer of atherosclerosis and thus triggers the manifestation of various cardiovascular events and pathologies. Among them are ischemic heart attacks and ischemic stroke. However, endothelial dysfunction is linked to hypertension, ischemia–reperfusion injury, diabetes, and neurodegenerative processes [79].

### Hcy-induced endothelial dysfunction

The endothelium has a variety of functions apart from adjusting the tone (dilation/contriction) of blood vessels. Any deviation from normal function of the endothelium is defined as endothelial dysfunction. This systemic pathological status is the core in the process of atherosclerosis and CVDs [80]. The vascular dilatation as a response to shear stress of blood flow is dependent on the endothelium-derived relaxing factor − NO. Further, its potent vasodilatory activity also suppresses platelet aggregation, supposing the thrombotic potential of HHcy may be modulated by the impairment of NO release/effects. Consequently, the failure of endothelial-mediated vasodilatory activity characterized by shifting the vascular balance toward an abnormally constrictive, inflammatory, and prothrombombic state is regarded as one of the earliest manifestations of cardiovascular damage supporting the formation of atherosclerotic plaques [81].

Several clinical and preclinical studies support the role of HHcy in the pathophysiology of endothelial dysfunction and consequent CVDs. Ahmed et al. (2020) evaluated whether high serum Hcy levels are associated with coronary microvascular endothelial dysfunction (CMED). The study involved participants with angina pectoris and non-obstructive coronary artery disease. Results showed that increased serum Hcy levels in patients correlated with higher rates of an invasive diagnosis of CMED. The authors summarized that the correlation between high Hcy levels and adverse cardiovascular events might potentially be modulated by coronary endothelial dysfunction. However, based on this study, no causal link can be univocally established [82]. He et al. (2010) investigated the link between damaged coronary endothelial function and chronic HHcy patients (plasma level of Hcy > 15 μmol/l), and if so, whether this impaired endothelial function is caused by the suppressed function of endothelial NO synthase (eNOS). Results revealed that plasma level of Hcy negatively correlates with coronary flow velocity reserve, and chronic HHcy may induce the onset of coronary artery disease by causing the dysfunction of the coronary artery endothelium. The malfunction of eNOS caused by chronic HHcy in these patients may partly explain this pathology [83]. Other authors pointed to various pathophysiological mechanisms of HHcy impairing endothelium-mediated NO-dependent vasodilatation. Hcy post-translationally downregulates dimethylarginine dimethylaminohydrolase enzyme activity (the enzyme that degrades ADMA), causing asymmetric dimethylarginine (ADMA, an endogenous inhibitor of NO synthase) to accumulate and thus inhibit NO synthesis [84]. Liang et al. (2021) described that Hcy activates the epithelial sodium channel and consequently induces endothelial dysfunction via reactive oxygen species (ROS)/COX-2-dependent activation of SGK-1/Nedd4-2 signaling [85]. Additionally, Hcy induced a calcium-mediated disruption of dynamics and mitochondrial function in endothelial cells due to overexpression of the mitochondrial calcium uniporter and the IP3R-Grp75-VDAC complex in mitochondria-associated membranes [86].

Comprehensive clinical research demonstrates that elevated Hcy levels associated with endothelial dysfunction represent the predisposing factor for the ethiopathogenesis of atherosclerotic processes and hypercoagulability states, which strongly correlate with cardiovascular mortality, coronary artery disease, and stroke.

### HHcy and coronary artery disease

Jin et al. (2021) evaluated the correlation between elevated plasma Hcy and heart failure subjects. This meta-analysis revealed significantly elevated plasma Hcy levels in patients with heart failure compared to the control individuals [76]. Another retrospective study evaluated the predictive role of HHcy for obstructive coronary artery disease (CAD) in an Asian population. Multivariate logistic regression analysis showed an independent correlation of HHcy with obstructive CAD in both old (aged > 55 years) and young individuals (aged ≤ 55 years). HHcy demonstrated a higher sensitivity (93.1%), accuracy (90.0%), and specificity (86.1%) for obstructive CAD compared to non-obstructive CAD [87]. Sun et al. (2021) analyzed whether HHcy is associated with acute coronary syndrome (ACS) and the severity of coronary artery stenosis in young Chinese adults. Young ACS subjects showed a greater prevalence of HHcy when compared with non-CAD individuals. In addition, HHcy in young ACS patients was linked with the severity of coronary artery stenosis, characterized by increased prevalence of multi-vessel disease, reduced value of left ventricular ejection fraction, and ST-segment elevation myocardial infarction (STEMI) [88]. Another study investigated the linkage between vitamin D deficiency and serum Hcy levels with the extent of CAD. This correlation was significant only among individuals with hypovitaminosis D. These results indicated that a normal vitamin D status can suppress the deleterious effects of HHcy on coronary atherosclerosis. However, this hypothesis needs further investigation [89]. On the contrary to the above-mentioned data, a two-sample Mendelian randomization study was conducted, i.e., “coronary heart disease” and “acute myocardial infarction.” Study results did not indicate a causal linkage between mentioned diagnosis and plasma Hcy levels. They concluded that conflicting data might have raised residual confounding or reverse causation [90].

### HHcy-associated ischemic stroke

The meta-analysis of Huang et al. (2020) investigated the prognostic utility of Hcy in individuals with acute ischemic stroke (AIS) in terms of all-cause mortality, poor functional outcome, and recurrent stroke. Independently elevated Hcy levels were linked with an increased risk of all-cause mortality but not poor functional outcome and recurrent stroke in subjects with AIS [91]. Another study revealed a causal association between plasma Hcy levels and ischemic stroke (IS) induced by small artery occlusion. However, the authors did not find a linkage between other types of IS, transient ischemic attack, neurodegenerative disease, and elevated plasma Hcy levels [92]. A comprehensive meta-analysis by Chinese researchers evaluated whether elevated Hcy levels represent an independent marker of unfavorable outcomes in AIS subjects. Total 15.636 AIS patients were analyzed in seventeen studies. Elevated Hcy plasma levels were linked with poorer survival of subjects. Hcy levels were significantly lower in the healthy control patients than in the AIS group with an SMD of 5.11 and 95% CI (1.87–8.35). Significant links between higher Hcy levels and the subject’s survival were observed only in Caucasians and Asians [91]. In a meta-analysis of prospective cohort studies, Wu et al. (2020) assessed the quantitative dose–response link of plasma Hcy levels with IS and stroke. They analyzed 10 prospective cohort studies using 11.061 participants in this analysis. Hcy levels were linked with elevated risk of IS and stroke for the highest vs the lowest categories. The authors found a linear association between the Hcy level and stroke [93]. Another meta-analysis revealed that elevated Hcy plasma levels are associated with a higher risk for IS and recurrent strokes but Hcy had no distinct linkage with hemorrhagic strokes [94].

Despite above mentioned promising data, studies analyzing the prognostic role of Hcy levels in subjects with CAD and IS are still rare. Definitive conclusions on this issue will require further clinical studies and in-depth analyzes.

## Association of HHcy with complications in pregnancy

Altered Hcy levels are implicated in pregnancy complications such as preeclampsia (PE) [95] or eclampsia [96]. These are considered the most common severe complications of pregnancy [96] and the leading causes of morbidity and mortality among pregnant women and fetuses [97]. PE is characterized by defects of placentation associated with hypertension in women that were previously normotensive and proteinuria after 20 weeks of gestation [95, 96] or new onset of hypertension combined with hematological, renal, liver, or neurological complications [97]. The state characterized by seizures in PE is defined as eclampsia [95, 96]. Although the etiopathology of PE is not fully understood, it is essential to identify risk factors to prevent PE development. Maternal concentrations of Hcy, folate, and vitamin B12 are investigated and evaluated in the development of PE. However, these efforts are not yet translated into clinical intervention [98]. Endothelial dysfunction is considered central in the pathophysiology of PE. During HHcy, Hcy auto-oxidizes to produce ROS that inactivates NO and thrombomodulin resulting in endothelial damage and dysfunction. Also, Hcy interferes with the fibrinolytic system, contributing to the pathophysiology of PE and eclampsia [96].

Nevertheless, maternal and cord micronutrients are frequently altered in women with PE or other pregnancy complications [99, 100]. A control case study showed pregnant women with HHcy possess a 7.7-fold risk for PE compared with normal controls [101]. More recently, higher maternal plasma Hcy levels were observed in women with PE compared with normotensive control (NC) women from early pregnancy, starting from the 16th week of gestation, until delivery accompanied by higher vitamin B12 levels. The study suggests the potential benefit of the Hcy analysis early in pregnancy before PE progression [95]. Also, Pisal et al. (2019) proved higher maternal Hcy levels at delivery accompanied by an increased level of vitamin B12 and folate in PE groups compared with NC. However, an increased maternal and cord Hcy was observed only in the term but not the pre-term PE group. The authors observed a positive association between maternal plasma Hcy and systolic and diastolic blood pressure in the whole group [99]. Further, HHcy is associated with PE and eclampsia; higher concentration of Hcy in eclampsia compared with preeclampsia indicates its relation to the severity of the disorder [96].

In addition, maternal HHcy is related to other pregnancy complications, such as pre-term birth, low body weight [97], placental abruption, recurrent pregnancy loss, or neural tube defects (NTD) of the newborn [96, 102]. Indeed, increased Hcy and decreased vitamin B12 were observed in mothers of neonates with NTD and in neonates with NTD [103]. Similarly, Felkner et al. (2009) demonstrated an association of high serum Hcy levels with pregnancies affected with NTD, even when serum red blood cell folate and B12 were high. These results suggest Hcy as an independent NTD factor [104]. Further, HHcy and oxidative stress were reported for women at risk of abortion or pre-term birth [102]. Overall, altered Hcy levels are crucial in PE, eclampsia, or other complications associated with the pregnancy or the newborn.

Hcy is metabolized to Met with vitamin B12 as a co-factor and folate as a co-substrate, while their deficiency is associated with an increased Hcy level [99]. However, above discussed results do not always support the notion of higher Hcy accompanied by reduced vitamin B12 and folate [99]. Indeed, low serum vitamin B12, folate, or RBC folate are incompatible with low or moderate Hcy levels but result in high Hcy levels. Nevertheless, elevated Hcy can occur concurrently with high serum vitamin B12, folate, or RBC folate, potentially due to deficiency of other metabolites causing high Hcy [104], poor cellular uptake of vitamin B12, or defects in Met synthase [99].

## Association of Hcy-axes with oxidative stress and inflammation

Elevated Hcy is related to various pathologies, while many of them are also associated with oxidative stress [105] or inflammation [106]. Redox state disbalance and oxidative stress are suggested as primary mechanisms associated with pathogenesis related to HHcy. ROS generation occurs during oxidation of the free thiol group of Hcy during its binding either with plasma proteins (such as albumin) or with other low-molecular plasma thiols or another Hcy molecule. Some of the proposed mechanisms of oxidative stress induced by Hcy include auto-oxidation of Hcy [107], inhibition of the expression or activity of antioxidant enzymes [107, 108], disruption of extracellular superoxide dismutase (SOD) from endothelial surface, or NO synthase-dependent superoxide anion generation [107]. Therefore, an elevated level of Hcy is relatively well explored in association with oxidative stress [109], and Hcy-associated ROS promote lipid peroxidation resulting in oxidative damage of cellular molecules [108]. Indeed, elevated Hcy levels are linked to de novo and recurrent cardiovascular events promoting an oxidant state in vascular cells and tissues. Accordingly, an increase in Hcy is considered a risk factor for CVD, including CAD [110]. Lipid peroxidation and oxidative stress demonstrated by increased Iso-P (8-isoprostane-prostaglandin F2) was observed in CAD patients with increased tHcy. An increased plasma intercellular adhesion molecule 1 (ICAM-1) and serum amyloid A (S-AA) in patients with high plasma tHcy suggest an association between hyperhomocysteinemia and low-grade inflammation [111]. In addition, the crucial role of oxidative damage in the development of CVD in postmenopausal women is associated with decreased oestrogen availability accompanied by increased oxidative stress. Indeed, recent study demonstrated that postmenopausal women are affected by oxidative stress that is independently related to the level of Hcy [112]. Also, Hcy was demonstrated to induce oxidative stress in young adult central retinal vein occlusion [109]. Except CVD, altered level of Hcy contributes to the redox imbalance and increased oxidative stress associated with the generation of ROS in other cell types such as neuronal, endothelial, glial cells leading to neurological disorders [113]. High concentration of Hcy is related to cognitive decline, AD, and dementia with oxidative stress suggested to play a crucial role. In fact, higher plasma Hcy and lower antioxidant level were observed in AD patients when compared with control [114]. Also, higher levels of oxidative stress was found to be accompanied by increased Hcy in patients with panic disorder when compared with healthy individuals [115].

Additionally, experimental and human models highlight the association between inflammation and HHcy. Pathogenic levels of Hcy affect inflammatory determinants such as adhesion molecules, endothelial dysfunction, oxidative stress, leukocyte adhesion, or reduced NO bioavailability [106]. Therefore, Hcy contributes to the conditions associated with inflammation, including cardiovascular or neuronal dysfunctions [106, 115, 116]. Further, increased Hcy is considered a risk factor for developing CVD and atherosclerosis in patients with rheumatoid arthritis. Yang et al. (2015) observed an increase in Hcy and associated immunological-inflammatory and metabolic markers in rheumatoid arthritis patients suggesting these markers’ potential role in assessing CVD risk in rheumatoid arthritis [117]. In addition, Hcy induced inflammation in the mouse retina, brain, and cultured human monocytes (U837). To this end, mild HHcy led to increased brain pro-inflammatory cytokines such as tumor necrosis factor-alpha (TNF-α), interleukin (IL)-1β, IL-6, and the chemokine monocyte chemotactic protein-1 (MCP-1) in Wistar rats [116]. Furthermore, Hcy treatment resulted in the elevation of pro-inflammatory and decrease of anti-inflammatory cytokines in a human retinal pigmented epithelial cell line (ARPE-19). Pro-inflammatory cytokines were also observed in human primary retinal endothelial cells (HRECs) treated with Hcy. These results support the role of Hcy-induced inflammation in the dysfunction of blood-retinal barriers and blood–brain barrier and pathogenesis of diabetic retinopathy, age-related macular degeneration, and AD [106]. Last but not least, altered Hcy levels contribute to other conditions associated with inflammation, including diabetes mellitus or chronic kidney disease; elevated Hcy levels are observed in inflammatory diseases such as inflammatory bowel disease and psoriasis [106, 116]. Furthermore, a recent study highlights the association between Hcy, bone mineral density, and inflammation in postmenopausal osteoporosis [118].

In conclusion, the crucial role of Hcy in oxidative damage and inflammatory responses needs to be precisely evaluated in the management of CVD and neuronal dysfunctions or other pathologies affected by Hcy-mediated oxidative stress and inflammatory conditions.

## Hcy and neurological disorders

Recent clinical studies strongly suggest that an elevated level of Hcy is an independent risk factor for neurological disorders [119]. Additionally, the effects of a disbalance of plasma Hcy are observed in several medical conditions, including PD [120], dementia [121], AD [122], or multiple sclerosis [123]. This section describes the relation between Hcy and the pathologies mentioned above and analyzes the potential role of Hcy as a possible predictive factor for these neurological conditions.

PD is characterized by the loss of striatal dopaminergic neurons (motoric manifestation of PD) and nondopaminergic neurons (non-motoric manifestation of PD) [124]. Levodopa (precursor of dopamine) is the most used medication to treat PD [125]. Long-term intake of levodopa leads to an increase in Hcy levels and subsequent to the progression of diseases connected to the onset of neuropsychiatric symptoms and the concomitant development of comorbidities (e.g., vascular disease). The formation of Hcy is caused by the O-methylation of levodopa which is catalyzed by the catechol-O-methyltransferase (COMT) [126]. Administration of COMT inhibitors effectively reduces Hcy but obtained data of several studies that focused on inhibitors’ effect on levodopa-induced HHcy are ambiguous [120, 127].

Dementia is a progressive cognitive decline that reduces a person’s ability to function independently [128]. An elevated level of Hcy is an independent risk factor associated with dementia [121]. Increased Hcy levels promote dementia development by disturbing the methylation or increasing the redox stress resulting in neuronal death [119, 129].

AD represents the most common form of dementia, characterized by neuritic plaque and neurofibrillary tangles [130]. A high concentration of Hcy is a risk factor for AD [122]. The association between Hcy and AD was intensely investigated, resulting in a better understanding of the different mechanisms by which Hcy contributes to its pathogenesis. Hcy can contribute to a disbalance in the neurological system resulting in AD through oxidative stress due to the generation of reactive oxygen species or suppression activity of antioxidants [122, 131]. Another way Hcy modulates cascades associated with AD involves demethylation of promoters (BACE-1 and Presenilin 1), leading to an elevated level of amyloid beta-peptide [132, 133]. Further, Hcy can cause cerebrovascular impairments associated with cognitive deficits, as demonstrated in Hcy-induced cerebrovascular disturbance in mice [134]. In addition, Hcy can affect amyloid beta-peptide and tau protein metabolism and thus accelerate changes that may result in AD [135, 136].

Multiple sclerosis is a chronic autoimmune-mediated inflammatory neurological disorder that affects the central nervous system [137]. A meta-analysis evaluating the correlation between Hcy and multiple sclerosis identified a significant increase in serum Hcy of patients [138]. Data indicate that elevated levels of blood Hcy may contribute to the disease’s pathogenesis.

A broader understanding of the roles of Hcy in neurological disorders and underlying mechanisms by which Hcy contributes to their progression might result in promising strategies to decrease the global incidence of mentioned neurological conditions.

## Association of Hcy-axes with cancer development and progression

Impaired plasma Hcy level is closely related to malignant processes [139]. Recent evidence revealed an elevated level of plasma Hcy in a cohort of patients with different cancer types, including breast [140], colorectal [141], ovarian [142], or lung [143]. A high level of Hcy is associated with venous thromboembolism, one of the leading causes of death in cancer patients [144]. Patients with an advanced stage of cancer manifest both HHcy and venous thromboembolism. On the other hand, patients with early-stage cancer have a low plasma Hcy, and venous thromboembolism is absent [139, 145]. In general, HHcy is a risk factor for venous thromboembolism after chemotherapy or surgery treatment [140, 146]. Patients undergoing surgery have an increased risk of venous thrombosis.

Similarly, the application of chemotherapy increased the risk of venous thromboembolism due to an increased level of Hcy [140]. Unfortunately, the exact mechanisms behind the relationship between elevated plasma Hcy and thromboembolism are not well understood. Hcy can act as a pro-oxidant that contributes to the generation of free radicals; thus, a high level of Hcy promotes endothelial cells’ oxidative damage and affects their physiological function [147]. Furthermore, Hcy can form a Hcy thiolactone acting as a reactive intermediate that creates covalent adducts with amino acids (lysine or arginine) residues in proteins leading to protein aggregation [148]. Subsequently, accumulating insoluble protein aggregates in the heart and blood can disturb normal heart function and physiology [139].

The Hcy detoxification pathway involves many enzymes participating in Hcy metabolism. Therefore, it is not surprising that mutations in this enzymatic machinery contribute to cancer development [149]. Different mutations and polymorphisms in specific genes, including *MTHFR*, *MTRR*, *MTR*, *MTHFD*, *BHMT*, *TCN 2*, *CBS*, and *TYMS* were identified in thrombosis or NTD [150, 151]. Specific polymorphisms connected to cancer were detected in *MTHFR*. Meta-analysis of 19 260 patients and 23 364 controls revealed that the C677T variant is the most common in ovarian and breast cancer patients. This polymorphism significantly increases the risk of breast and ovarian cancer in Asians. Interestingly, C677T represents increased risk factors for breast cancer in Caucasians, but there was no significance between C677T and ovarian cancer in Caucasoid populations [152]. Furthermore, other studies observed a significant association between *MTHFR* C677T polymorphism and esophageal cancer [153], neck cancer [154], and lung cancer [155]. Another *MTHFR* polymorphism, A1298C, is associated with breast [156] and bladder [157] cancer susceptibility. Molecular analysis of the *MTRR* gene identified A66G polymorphism significantly associated with lung cancer in a Turkish population [158]. Also, the A66G variant was determined as a risk factor for colorectal cancer in a Japanese population [159]. *MTR* A2756G polymorphism was recognized as a risk factor for breast [160], head, and neck squamous cell carcinoma [161], or acute lymphoblastic leukemia [162]. *MTHFD1* G1958A polymorphism is described as a risk factor for head and neck cancer development [163]. *BHMT* is another essential gene contributing to Hcy metabolism. Substitution G to A on position 742 represents (G742A) a polymorphism raising risk of HNSCC.

As previously mentioned, folate has an inverse relation with Hcy. Folate contributes to nucleotide biosynthesis, Met biosynthesis as well as cellular methylation reactions [164]. In addition, folate is essential for converting deoxyuridine monophosphate (dUMP) to thymidylate catalyzed by thymidylate synthase (TYMS) [165]. This reaction involves the transfer of the methyl group from 5,10-methylenetetrahydrofolate, which is derived from folate. Under conditions, when methyl donor 5,10-methylenetetrahydrofolate is absent due to folate limitation, dUMP accumulates, which results in excessive uracil incorporation into DNA instead of thymine. During the physiological condition are incorrectly incorporated uracils removed by DNA glycosylase. However, the DNA glycosylase repair system fails due to low folate concentration and high concentration of Hcy, resulting in chromosomal damage and subsequent promotion of carcinogenesis [166].

An elevated level of Hcy also correlates with alteration in DNA methylation machinery, which plays a crucial role in regulating gene expression [167]. As mentioned above, an increased Hcy level is related to folate concentration [168]. DNA methylation as an epigenetic mechanism requires a methyl donor, SAM, acquired from Met through the enzymatic reaction catalyzed by S-adenosyl synthetase. Subsequently, SAM is used as a methyl donor in DNA methylation reactions catalyzed by DNMTs [169]. Disbalance in SAM production due to the limitation of 5,10-methylenetetrahydrofolate (key substrate responsible for Met regeneration) results in impaired DNA methylation leading to extensive hypomethylation of the genome [139, 170]. Global hypomethylation represents a hallmark of various cancer that contributes to destabilizing chromosomal integrity and thus promotes carcinogenesis [171–173].

In summary, an elevated level of Hcy can contribute to cancer initiation, promotion, and progression. The specific polymorphisms in genes contributing to Hcy metabolism or diet deficiency in folate, vitamin B6, or cobalamin directly correlate with the Hcy level’s disbalance. Further, administration of drugs, including laxatives, birth control pills, or immunosuppressive drugs, is associated with elevated Hcy and subsequent folate reduction [174, 175]. A better understanding of mechanisms behind the role of Hcy in carcinogenesis will bring new opportunities in cancer-related research and accelerate novel therapies targeting disbalances in Hcy metabolism.

## Hcy metabolism-associated eye disorders

As precisely described, an elevated level of Hcy affects the cardiovascular system [176] and increases the risk of acute ischemic stroke [107], pregnancy complications [177], impaired wound healing [178], neurological disorders [119], cancer development, and cancer-associated complications [139]. Further, current data identified a cross-connection between altered Hcy levels and ocular diseases such as retinopathy, cataract, maculopathy, optic atrophy, pseudo-exfoliative glaucoma, and retinal vessel atherosclerosis [179].

Diabetic retinopathy (DR) is a microvascular complication and the most common cause of blindness in people under 65 worldwide [180, 181]. Tawfik et al. (2019) measured Hcy levels in serum, vitreous, and retina of patients with diabetes. Additionally, they evaluated Hcy levels in serum and retina of animal models representing diabetes type 1 and type 2. They revealed an elevation of Hcy in serum, vitreous, and the retina in patients and animal models. Furthermore, intravitreal injection of Hcy caused retinal changes in animals. These changes of the retina were more severe in diabetic mice than in wild type. Hcy can be used as a promising biomarker in patients with DR [181]. To this end, intravitreal injection of Hcy thiolactone, an intramolecular thioester of Hcy, resulted in degeneration of photoreceptors in mice, which could lead to retinopathies [182].

Prolonged exposure to Hcy can result in a cataract formation in the eye lens [183]. An increased plasma Hcy level is associated with a higher prevalence of posterior subcapsular cataracts in patients [184]. A notable association between *MTHFR* polymorphisms and risk of age-related cataracts was investigated by Wang et al. (2015). They identified a correlation between variants of the *MTHFR* gene, which could be a risk factor for age-related cataracts. These polymorphisms can modulate MTHFR enzyme activity and subsequent Hcy levels [185].

Age-related macular degeneration is the most common permanent vision loss affecting people aged 60 and older [186]. A correlation between age-related macular degeneration and increased levels of Hcy was documented in a meta-analysis [187]. In addition, elevated levels of Hcy thiolactone and Hcy are associated with the pathogenesis of age-related macular degeneration [188].

The pseudo-exfoliation syndrome (PEX) is a systematic, age-related disorder characterized by the accumulation of fibrinous material in the eye (most notably within the anterior chamber of the eye) [189]. Additionally, PEX is the leading cause of pseudo-exfoliation glaucoma (secondary open-angle glaucoma) development [190]. A recent study revealed an association between the elevated level of plasma Hcy and pseudo-exfoliation glaucoma. Acquired data identified significantly increased plasma Hcy in patients with pseudo-exfoliation glaucoma compared to patients with primary open-angle glaucoma and healthy controls [191]. The retinal artery occlusive disease (ROA) is another pathological condition of the eyes. It is defined as a loss of vision due to blockage of the retinal artery [192]. A relationship between ROA and elevated levels of Hcy was found in a meta-analysis of cohort studies. Results indicated that an increased level of plasma Hcy could act as an independent risk factor associated with ROA [193].

In conclusion, a direct correlation between HHcy and ocular disorders predicts Hcy as a promising biomarker. A more in-depth investigation into molecular secrets behind the role of Hcy in eye diseases can accelerate current research, bring new therapeutical strategies, and thus improve the overall life quality.

## HHcy as a risk factor of impaired healing

HHcy is considered to be a risk factor of delayed and impaired healing. To this end, Type 2 diabetic patients with chronic bilateral, medial ankle venous ulcers, and elevated serum Hcy level have been demonstrated as not responding to treatment with a topical human fibroblast-derived dermal substitute; however, after normalization of Hcy level by folic acid, vitamin B6, and B12, the reapplication of the same treatment led to an improved healing process [194]. To this end, Hcy-lowering therapy by application of folic acid accelerates wound healing in patients with chronic venous ulceration that underwent compression therapy and surgical procedures. HHcy patients that received basic treatment and were administered folic acid (1–2 mg/day for 12 months) had a higher healing rate than non-HHcy patients who received only basic treatment [195]. For example, in a 26-year-old man with chronic leg ulcers, the administration of B vitamins (B1, B2, B6, and B12), trimethyl-glycine, mecobalamine, folic acid, and povidone-iodine dressings with culture-directed antibiotic therapy was associated with improved healing of ulcers over 1 month [196]. Another example is a male patient (60 years old) with HHcy and MTHFR heterozygosity in segments C677T and A1298C had deteriorating healing of leg ulcers that can lead to the evolution of verrucous elephantiasis nostra. Six months of treatment with vitamin B complex and oral folic acid improved the Hcy level and healed the dermatological lesions [197].

Several preclinical studies also focused on the association between HHcy and impaired healing. Elevated Hcy level was associated with the impaired/slow downed femoral fracture healing in mice on Hcy-supplemented diet (*n* = 12) compared to mice on standard diet (*n* = 13) [198]. On the contrary, an in vivo study revealed that folate and vitamin B12 deficiency in diet did not affect bone repair in mice [199]. To this end, HHcy inhibited tibial fracture healing in rats by suppressing PI3K/AKT signaling pathway and enhanced apoptosis and level of pro-inflammatory TNF-α [200]. Interestingly, higher Hcy levels and decreased vitamin B12 were observed in Hcy-treated rats than in control rats. In Hcy-treated rats, the elevated Hcy level also reduced the bone’s blood flow, which contributed to compromised bone biomechanical properties [201].

Furthermore, patients with inflammatory bowel disease have a higher risk of HHcy due to vitamin B deficiency. The administration of B vitamins (B6, B9, and B12) was associated with the worsened colitis in rodents due to increased serum Hcy level related to the absence of injury-induced elevation of H_2_S synthesis. However, the administration of IL-10 with an ability to increase H_2_S synthesis ameliorated the severity of colitis, reduced serum Hcy levels, and inflammation, thereby promoting healing [202].

As was mentioned above, several studies focused on the association between HHcy and impaired healing. To mitigate the adverse effects of HHcy to wound healing, the supplementation of cofactors (B vitamins, folic acid) or other agents (IL-10) seems to be perspective in the treatment of several diseases, especially in type 2 diabetic patients, patients with various types of ulcers, fractures, or inflammatory bowel diseases. This supplementation has the potential to significantly decrease the Hcy level and facilitate/accelerate wound healing. Table 1 provides an overall summary of the results of the above-mentioned studies of which HHcy were associated with various complications, including oxidative stress, inflammation, CVD, pregnancy, neurological and eye disorders, cancer, and healing.

**Table 1:** Overall summary of preclinical and clinical studies focused on HHcy and associated impairments

Disease	Study design	Study participants	Results	Ref
Mitochondrial dysfunction				
Acute myocardial ischemia–reperfusion injury	Plasma	Male Sprague–Dawley rats and H9C2 (2–1) cells	Elevated plasma Hcy-induced mitochondrial dysfunction and oxidative stress through increased cytochrome *c* release, stimulation of ROS production, and ERK1/2 signaling pathway	[66]
Elderly frailty, skeletal muscle weakness, and fatigability	-	C57 and CBS + / − mice	HHcy caused mitochondrial dysfunction through reduced dystrophin levels along with a decrease in mtTFA and its regulator NRF-1	[67]
Cerebral infarction-related disease	Hcy-treated ischemic brains	Male Sprague–Dawley rats	Elevated Hcy level inhibited the enzymatic activity of mitochondrial complex I–III that was associated with higher cytochrome *c* release; Hcy increased 8-Hydroxy-2′-deoxyguanosine content and mitoStat3 protein phosphorylation	[68]
Vascular injury	Hcy treatment	Human umbilical vein endothelial cells	Hcy treatment induced mitochondrial apoptosis through increased NOX4 expression and intracellular ROS production and decreased Bcl-2/Bax ratio and MMP, resulting in cytochrome *c* release and caspase-3 activation	[69]
PD	Hcy treatment	Male Sprague–Dawley rats	Hcy reduced activity of mitochondrial complex-I and caused oxidative stress associated with increased production of hydroxyl radicals, reduced glutathione level, and enhanced activity of antioxidant enzymes such as superoxide dismutase and catalase	[70]
Cellular senescence and aging				
Cellular senescence	Hcy treatment	Endothelial cells	Hcy shortened telomeres through DNA hypomethylation of human telomerase reverse transcriptase and increased acidic β-galactosidase; Hcy upregulated the markers of cellular senescence p16, p21, and p53; the administration of folic acid or SAM could reverse mentioned effect	[72]
Cellular senescence and atherosclerosis	Chronic exposure to Hcy	Endothelial cells	Hcy accelerated the rate of cellular senescence through a redox pathway suggesting that oxidative stress could increase the production of vascular cell senescence proven by increased expression of two surface molecules such as intracellular adhesion molecule-1 (ICAM-1) and plasminogen activator inhibitor-1 (PAI-1)	[73]
Coronary heart disease	Exposure to Hcy	EPC	Hcy decreased proliferation and increased EPC senescence through diminished telomerase activity and Akt phosphorylation; the treatment with atorvastatin revealed the preventive effect against Hcy-induced senescence of EPC	[74]
Cardiovascular diseases				
Coronary microvascular endothelial dysfunction	Serum	Patients (*n* = 1418) with angina pectoris and non-obstructive coronary artery disease	Increased serum Hcy levels in patients correlated with higher rates of an invasive diagnosis of coronary microvascular endothelial dysfunction	[82]
CAD	Plasma	Damaged coronary endothelial function and chronic HHcy patients (*n* = 71)	Plasma level of Hcy negatively correlates with coronary flow velocity reserve; chronic HHcy may induce the onset of coronary artery disease by causing the dysfunction of the coronary artery endothelium that could be related to the malfunction of eNOS	[83]
CVD associated with endothelium-dependent vasodilatation	DDAH Binding Assay	Primary bovine aortic endothelial cells	Hcy post-translationally downregulates dimethylarginine dimethylaminohydrolase enzyme activity causing asymmetric dimethylarginine to accumulate and thus inhibit NO	[84]
Endothelial dysfunction	Plasma	C57BL/6 J mice; L-methionine in a chow diet for 4 weeks to establish the HHcy; Human umbilical vein endothelial cells	Hcy activates the epithelial sodium channel and consequently induces endothelial dysfunction via reactive oxygen species (ROS)/COX-2-dependent activation of SGK-1/Nedd4-2 signaling	[85]
Endothelial dysfunction	-	Human umbilical vein endothelial cells	Hcy induced a calcium-mediated disruption of dynamics and mitochondrial function due to overexpression of the mitochondrial calcium uniporter and the IP3R-Grp75-VDAC complex in mitochondria-associated membranes	[86]
Heart failure	Plasma	Heart failure patients in (*n* = 5506)	Elevated plasma Hcy levels in patients with heart failure compared to the control individuals	[76]
CAD	Serum	Patients (*n* = 2987) of Asian population; non-obstructive CAD group (*n* = 1172) and obstructive CAD group (*n* = 1815)	Correlation of HHcy with obstructive CAD in both old (aged > 55 years) and young individuals (aged ≤ 55 years); HHcy demonstrated a higher sensitivity (93.1%), accuracy (90.0%), and specificity (86.1%) for obstructive CAD compared to non-obstructive CAD	[87]
CAD, coronary acute syndrome, and coronary artery stenosis	Serum	Young Chinese adults (*n* = 1.103, 18–35 years old); CAD patients (*n* = 828) and non-CAD patients (*n* = 275)	Young coronary acute syndrome patients showed a greater prevalence of HHcy when compared with non-CAD individuals; HHcy in young patients was linked to the severity of coronary artery stenosis, characterized by increased prevalence of multi-vessel disease, reduced value of left ventricular ejection fraction, and ST-segment elevation myocardial infarction	[88]
CAD	Serum	Patients (*n* = 3150) undergoing coronary angiography	Normal vitamin D status can suppress the deleterious effects of HHcy on coronary atherosclerosis	[89]
CAD and myocardial infarction	Plasma	Coronary heart disease patients (*n* = 184,305) and acute myocardial infarction patients (*n* = 181.875)	Results did not indicate a causal linkage between CAD or myocardial infarction and plasma Hcy levels	[90]
Acute ischemic stroke	Serum	Acute ischemic stroke patients (*n* = 15,636)	Elevated Hcy levels were linked with an increased risk of all-cause mortality but not poor functional outcome and recurrent stroke in subjects with acute ischemic stroke	[91]
Ischemic stroke	Plasma	Meta-analysis	Causal association between plasma Hcy levels and ischemic stroke induced by small artery occlusion	[92]
Acute ischemic stroke	Plasma	Acute ischemic stroke patients (*n* = 15.636)	Elevated Hcy plasma levels were linked with poorer survival of subjects; significant links between higher Hcy levels and the subject’s survival were observed only in Caucasians and Asians	[91]
Stroke	Plasma	Stroke patients (*n* = 11.061)	Hcy levels were linked with elevated risk of ischemic stroke (RR = 1.54, 95% CI 1.21–1.97, *I*^2^ = 36.4%) and stroke (RR = 1.58, 95% CI 1.25–2.00, *I*^2^ = 39.5%)	[93]
Stroke	Plasma	Ischemic stroke patients (*n* = 13.284)	Elevated Hcy plasma levels are associated with a higher risk for IS and recurrent strokes but Hcy had no distinct linkage with hemorrhagic strokes	[94]
Pregnancy complications				
PE	Plasma	PE (*n* = 32) and controls without pregnancy complications (*n* = 64)	Pregnant women with HHcy have a 7.7-fold risk for PE vs normal controls (Hcy and folate higher in PE vs control in third trimester)	[101]
	Maternal blood (plasma) collected three times during pregnancy: 16th–20th weeks (T1), 26th–30th weeks (T2), at delivery (T3)	NC (*n* = 126) and PE (*n* = 62)	Higher maternal plasma Hcy level in women with PE vs normotensive women at all three time points; maternal plasma vitamin B12 higher in PE vs NC at T2	[95]
	Maternal and cord blood collected at delivery	NC (*n* = 450) and PE (*n* = 350); PE women delivering at term (*n* = 224) and pre-term (*n* = 126)	Maternal and cord Hcy higher in PE vs NC (Hcy higher in the term PE group); positive association of maternal plasma Hcy with systolic and diastolic blood pressure (whole cohort)	[99]
Eclampsia, PE	Serum	Healthy pregnant controls (*n* = 136), PE pregnant (*n* = 84), and eclamptic pregnant (*n* = 120)	Serum Hcy increased in PE and eclampsia vs control; Hcy raised more in eclampsia vs PE	[96]
PE, pre-term birth, low birth weight	Serum	Pregnant women with adverse outcome (*n* = 563) and controls (*n* = 600)	Upper-quartile Hcy levels associated with PE, preterm birth, and low birth weight vs lower-quartile	[97]
NTD	Plasma	Mothers with first NTD child or with a history of NTD child in the family (*n* = 96), neonates with spina bifida (*n* = 126), mothers with normal previous and current pregnancies (*n* = 84), and control neonates with no defects (*n* = 87)	Increased serum Hcy and decreased vitamin B12 in mothers with neonates with NTD and in neonates with NTD	[103]
NTD	Serum	Case women (*n* = 103) — diagnosis of anencephalous, spina bifida, or encephalocele and control women (*n* = 139) — delivering normal live births	High serum Hcy associated with NTD-affected pregnancies (even when serum B12 and RBC folate is high)	[104]
Abortion, pre-term birth	Serum	Patients with risk of abortion (*n* = 18), patients with pre-term birth (*n* = 22), and healthy pregnant controls (*n* = 14)	Higher level of Hcy and MDA (marker of oxidative stress) in women with risk of abortion or with pre-term birth	[102]
Oxidative stress and inflammation				
CAD	Blood	Patients with ischemic heart disease (*n* = 93)	Increased Iso-P (marker of lipid peroxidation and oxidative stress in CAD patients with increased tHcy; increased plasma ICAM-1 and S-AA in CAD patients with high plasma tHcy → association between homocysteinemia and low-grade inflammation	[111]
CVD in postmenopausal women	Serum	Healthy pre- (*n* = 223) and postmenopausal (*n* = 118) Omani women	Postmenopausal women affected by oxidative stress (independent relation to Hcy level)	[112]
Young adult CRVO	Plasma	Young adult CRVO (*n* = 23) and controls (*n* = 54)	Hcy induce oxidative stress	[109]
AD	Serum	AD patients (*n* = 143) and controls (*n* = 1553)	Higher plasma Hcy and lower antioxidant level observed in AD patients vs control	[114]
Panic disorder	Blood	Panic disorder patients (*n* = 60) and healthy individuals (*n* = 60)	Increased oxidative stress accompanied by elevated Hcy in patients with panic disorder vs healthy individuals	[115]
Rheumatoid arthritis	Serum	rheumatoid arthritis patients (*n* = 50) and controls (*n* = 50)	Increased Hcy and associated immunological-inflammatory and metabolic markers in rheumatoid arthritis patients	[117]
Pro-inflammatory cytokine level	Brain, heart, serum of rats	Mild hyperhomocysteinemia induced in Wistar rats by Hcy administration (0.03 μmol/g of body weight) twice a day	Hcy induced inflammation in mouse retina, brain, and cultured human monocytes (U837); mild HHcy increased brain pro-inflammatory cytokines as TNF-α, IL-1β, IL-6, and MCP-1 in Wistar rats	[116]
Inflammation in the dysfunction of blood-retinal barriers and blood–brain barrier and pathogenesis of diabetic retinopathy, age-related macular degeneration, and AD		Mice with HHcy (tissue lysates isolated from the brain hippocampal area), HRECs, human retinal pigmented epithelial cell line (ARPE-19)	Hcy increased pro-inflammatory and decreased of anti-inflammatory cytokines in ARPE-19; pro-inflammatory cytokines observed HRECs treated with Hcy	[106]
Postmenopausal osteoporosis	Serum	Postmenopausal women (*n* = 252)	Hcy associated with bone mineral density, and inflammation in postmenopausal osteoporosis	[118]
Cancer				
BC	Plasma	BC patients (*n* = 35)	Increased level of plasmatic Hcy and vitamin B12 during chemotherapy while folate and platelets were decreased	[140]
CC and BC	Plasma	CC and BC patients (*n* = 47)	Increased level of Hcy in the cancer patients characterized by low-grade inflammation	[141]
LC	Plasma	LC patients (*n* = 37) and controls (*n* = 26)	Increased level of total Hcy, lower level of total glutathione, and folate compared to control; no significance was observed between SCLC and NSCLC patients	[143]
Eye disorders				
Diabetic retinopathy	Serum	Diabetic patients and mice models of diabetes	A higher level of Hcy was detected in serum, vitreous, and retina of patients and mice	[181]
Age-related macular degeneration	Plasma	Age-related macular degeneration patients (*n* = 16) and 16 matched controls	Increased level of Hcy with elevated Hcy- thiolactone, thiobarbituric acid reactive substance (TBARS); the glutathione level was reduced	[188]
Pseudoexfoliation glaucoma	Plasma	PEXG patients (*n* = 36), POAG patients (*n* = 40), and 40 controls (*n* = 40)	Plasmatic Hcy was increased in PEXG group compared to POAG group	[191]
Neurological disorders				
PD	Plasma	PD patients treated by L-dopa (*n* = 26), PD patients treated by L-dopa + COMT-I (*n* = 20), healthy controls (*n* = 32)	Increased level of plasma Hcy in PD patients. A significantly lower level of plasma Hcy in the group treated by L-dopa + COMT-I	[126]
Psychological symptoms of Dementia (BPSD) in AD	Serum	AD patients (*n* = 18) and healthy controls (*n* = 18)	Correlation between increased Hcy in serum and behavioral and psychological symptoms observed in patients with AD	[121]
AD	Hippocampal slices of rats	Male Sprague–Dawley rats injected by Hcy (400/1600 μg/kg/day). Rats (two groups) were fed with or without folate and vitamin B12	Supplementation of folate and vitamin B12 restored Hcy plasma level and antagonized the Hcy-induced tau hyperphosphorylation	[136]
Healing				
Chronic bilateral, medial ankle venous ulcers	Serum	A 79-year-old white male patient with type 2 diabetes mellitus, hypertensive CVD, chronic bilateral venous insufficiency, peripheral vascular disease, elevated fasting serum Hcy (14.9 μmol/L), and lower extremity neuropathy	Normalization of Hcy level by folic acid, vitamin B6, and B12; treatment with a topical human fibroblast-derived dermal substitute led to the wound healing within 4 weeks	[194]
Chronic venous ulceration	Plasma	HHcy patients with chronic venous ulceration (*n* = 54) that underwent compression therapy and surgical procedures; non-HHcy patients who received only basic treatment (*n* = 33)	Hcy-lowering therapy with folic acid (1–2 mg/day for 12 months) accelerates wound healing in patients that underwent compression therapy and surgical procedures	[195]
Chronic leg ulcers	Plasma	A 26-year-old man with chronic leg ulcers of eight months duration	Administration of B vitamins (B1, B2, B6, and B12), trimethyl-glycine, mecobalamine, folic acid, and povidone-iodine dressings with culture-directed antibiotic therapy improved healing of ulcers over 1 month	[196]
Leg ulcers	Serum	A male patient (60-year-old) with HHcy and MTHFR heterozygosity (C677T and A1298C)	Six months of treatment with vitamin B complex and oral folic acid improved the Hcy level and healed the dermatological lesions	[197]
Femoral fracture	Serum	CD-1 mice on Hcy-supplemented diet (*n* = 12), control mice on standard diet (*n* = 13)	Elevated Hcy level was associated with the impaired/slow downed femoral fracture healing in mice on Hcy-supplemented diet	[198]
Femoral fracture	Serum	Folate and vitamin B12 deficient diet in CD-1 mice (*n* = 14), control mice with equicaloric diet (*n* = 13)	Folate and vitamin B12 deficiency in diet did not affect bone repair in mice	[199]
Tibial fracture	Plasma	Sprague–Dawley rats: sham group (*n* = 12), tibial fracture group (*n* = 12), and HHcy + fracture group (*n* = 12)	HHcy inhibited tibial fracture healing by suppressing PI3K/AKT signaling pathway and enhanced apoptosis and level of TNF‑α	[200]
Osteoporosis	Plasma	Male Sprague–Dawley rats: wild-type group (*n* = 10) and an HHcy group (*n* = 12); Hcy was supplemented (0.67 g dl-Hcy/L drinking water) for 8–12 weeks	Higher Hcy levels and decreased vitamin B12 reduced the bone's blood flow, which contributed to compromised bone biomechanical properties	[201]
Inflammatory bowel disease	Serum	Male Wistar rats and C57BL/6 J homozygous IL-10–deficient mice; B vitamins deficient diet or control diet	Administration of IL-10 with an ability to increase H_2_S synthesis ameliorated the severity of colitis, reduced serum Hcy levels and inflammation, thereby promoting healing	[202]

Abbreviations: *AD*, Alzheimer’s disease; *BC*, breast cancer; *CAD*, coronary artery disease; *CC*, colorectal cancer; *CRVO*, central retinal vein occlusion; *CVD*, cardiovascular diseases; *Hcy*, homocysteine; *HHcy*, hyperhomocysteinemia; *HREC*, human primary retinal endothelial cells; *IL*, interleukin; *Iso-P*, 8-isoprostane-prostaglandin F 2; *LC*, lung cancer; *MCP-1*, chemokine monocyte chemotactic protein-1; *MDA*, malondialdehyde; *NC*, normotensive control; *NTD*, neural tube defects; *PD*, Parkinson’s disease; *PE*, preeclampsia; *PEXG*, pseudoexfoliation syndrome; *POAG*, primary open-angle glaucoma; *RBC*, red blood cell; *TNF-α*, tumor necrosis factor-alpha; *MTHFR*, methylenetetrahydrofolate reductase; *H*_*2*_*S*, hydrogen sulfide; *PI3K*, phosphoinositide 3-kinase; *AKT*; protein *kinase* B; *TNF-α*, tumor necrosis factor alpha; *NO*, nitric oxide; *eNOS*, endothelial NO synthase; *NOX4*, NADPH oxidase 4; *MMP*, mitochondrial membrane potential; *mtTFA*, mitochondrial transcription factor A; *NRF-1*, nuclear respiratory factor 1; *EPC*, endothelial progenitor cells

## Nutritional recommendations to prevent HHcy and associated pathologies

HHcy is associated with mutations of relevant genes or nutritional depletion of related vitamins [203]. A healthy diet rich in vitamins and antioxidants plays an essential role in the organisms, thus ensuring the proper Hcy metabolism function. This paragraph underlines the importance of low-Met diet, dietary intake rich in B vitamins, as essential cofactors for Hcy metabolism, their synthetic forms such as folic acid or betaine in patients with HHcy.

Whole-grain intake is inversely associated with Hcy in healthy men and women — the mean Hcy concentration was 17% lower in subjects with the highest whole-grain consumption compared with the lowest [204]. To reduce Hcy levels and associated deaths in patients, supplementation with the activated forms of B6, folate, B12, or betaine should be used [21]. Co-supplementation of folic acid and vitamin B12 exerts a synergistic effect in lowering blood Hcy [205]. Interestingly, the Mediterranean diet can also decrease Hcy levels. A 19% decline in Hcy level after 2 weeks happened after administration of a fiber-rich diet [206]. Dietary intake of vitamin B12 and folate is essential for Hcy metabolism. Therefore, deficiency in these vitamins can cause the HHcy and associated diseases. The intake of dairy and meat products is vital to achieving the daily B12 intake of 3.0 μg to prevent HHcy [50]. Vitamin B12 is commonly found in dairy and meat products [50], legumes, and green leafy vegetables [207]. Asparagus, beef liver, legumes, and egg yolk are rich in folate [208]. Furthermore, recommended daily intake of folate (400 μg) can also be achieved by the supplementation of folic acid, a synthetic version of folate with the ability to convert to folate by the body, that is important in vegetarians and older adults as the risk groups of folate deficiency [209]. A randomized clinical trial demonstrated the capacity of high-dose folic acid (5 mg/day) supplements administered throughout pregnancy to decrease Hcy concentrations at the time of delivery [210]. Similarly, high-dose folic acid supplement from 3 months before pregnancy until the entire pregnancy reduced recurrent PE [211].

Vegetables and fruits are rich in phytochemicals that exert many health benefits and thus play an essential role in preventing and treating chronic diseases such as CVDs [212]. The supplementation with genistein (5, 7-dihydroxy-3- (4-hydroxyphenyl)-4H-1-benzopyran-4-one) reduced plasma Hcy levels significantly. For this reason, genistein is considered as a potential substance for the prevention and treatment of CVDs and reduction of cardiovascular mortality [213]. Further, epigallocatechin-3-gallate (EGCG) prevents Hcy-induced apoptosis in endothelial cells by upregulation of SIRT1/AMPK and Akt/eNOS cell signaling. These data indicate that EGCG might have some benefits for HHcy-induced endothelial dysfunction and by this mechanism can prevent CVDs [214]. Also, curcumin demonstrated antagonistic activity to Hcy. In preclinical research, curcumin had protective effects against endothelial dysfunction via upregulation of eNOS expression and reduction of oxidative DNA damage in cardiomyocytes [215]. Furthermore, coffee intake of 1–3 cups/day containing polyphenols is linked to decreased levels of Hcy. Based on these data, it is rational that moderate coffee consumption has preventive effects against some cardiovascular risk factors [216].

Quercetin exerted protective effects on Hcy-induced oxidative stress in a rat model demonstrated through higher plasma levels of erythrocyte catalase, an enzyme of the antioxidant defense system, and decreased plasma malondialdehyde (MDA), a product of lipid peroxidation [108]. Similarly, the administration of melatonin and vitamin E could exert beneficial effects in preventing the effects of Hcy on plasma antioxidant enzymes, as demonstrated by the impeded decrease of plasma antioxidant enzyme activity in Hcy-treated male rats [217]. The citrus flavonoid hesperidin protects against HHcy by abrogating oxidative stress, endothelial dysfunction, and neurotoxicity in male Wistar rats [218]. As discussed above, current evidence supports the role of oxidative stress in AD [219], while increased Hcy and lower antioxidant levels are observed in AD patients [114]. However, a multicenter, randomized, double-blind controlled clinical trial stated that regular intake of polyphenols in the antioxidant beverage might be beneficial in the decrease of tHcy plasmatic concentrations in AD [219]. Quercetin also exerted protective effects on Hcy-injured human umbilical vein vascular endothelial cells (ECV304) by antioxidant and anti-inflammatory activity demonstrated through decreased MDA, endothelin release, and NF-κB and increased SOD activity, NO, and 6-keto-prostaglandin F1alpha release. These findings suggest the potential of quercetin as a preventive or therapeutic agent in CVD [220].

The modulation role of diet affecting specific molecular pathways associated with cancer is currently intensively studied. An observational study identified that high folate status led to a decreased plasma Hcy level and subsequently increased DNA methylation in colon tissue in a cohort of patients with colorectal cancer and colorectal adenoma. Their observation revealed a cross-connection between low folate intake and DNA hypomethylation with an increased risk of colorectal neoplasia [221]. Further, higher folate intake (folate has an inverse relation with Hcy) is associated with decreased risk of developing different tumors such as colorectal or esophageal cancer in the Uruguay population [222]. Notably, phytochemicals, naturally occurring non-nutritional compounds of plants, exert beneficial features for human health, including modulation of key molecular cascades associated with Hcy metabolism. Their additional intake is promising to prevent carcinogenesis but further research in cancer chemoprevention, mediated by phytochemicals, is needed [223].

The regular consumption of vitamins (i.e., B12) and other dietary supplements is associated with the prevention of numerous pathological conditions, including HHcy affecting normal ocular function. Vitamin B12 deficiency is a common cause of various health problems [224]. There is a correlation between the decreased vitamin B12 and increased age of the probands in both genders. Similarly, a low folate level is associated with an elevated level of Hcy in the elderly population. Daily dietary intake of vitamins B12 and folic acid was required for patients with glaucoma to reduce the level of Hcy [179].

Similar to the aforementioned pathological conditions, low dietary intake of vitamins, including B6 and B12, leads to increased Hcy levels, affecting the pathogenesis of various neurological disorders [225]. Several case–control and prospective cohort studies tried to confirm the association between low riboflavin, folate, and vitamins B6 and B12 intake with increased risk of PD. Interesting results showed periodic supplementation with folic acid leading to reduction of Hcy level in a cohort of patients with PD [226–228]. A lower level of vitamin B12 and a higher level of Hcy cause a reduction in mobility and more cognitive decline in a cohort of patients with PD [229].

Nutritional recommendations for maintaining normal Hcy levels are usually focused on patients with specific mutations in enzymes associated with Hcy metabolism. If the plasma Met concentrations exceed 800 μmol/L, a low-Met diet could be beneficial, especially in patients with Met adenosyltransferase I or III, SAH hydrolase, and adenosine kinase deficiencies [230]. In patients with CBS deficiency, the treatment with a low-Met diet and betaine is highly recommended to maintain plasma Hcy concentration below 120 μmol/L [231]. Table 2 summarizes the nutritional recommendations, their recommended doses for the prevention of HHcy, and associated impairments.

**Table 2:** Nutritional recommendations for targeted prevention of HHcy and associated systemic effects

HHcy associated with disease/complications	Supplement/diet	Recommend dose	Ref
Risk of diabetes and ischemic heart disease	Whole-grains	Median intake: 22.3 g/day	[204]
Risk of vascular disease and associated deaths	Folic acid + vitamins B12	Folic acid: 0.5–5 mg/day, vitamin B12: 0.5 mg/day	[205]
Risk of CVDs	Fiber-rich diet (a low-calorie, high-fiber, fruit-based nutrient-dense bar rich in vitamins, minerals, fruit polyphenolics, β-glucan, docosahexaenoic acid)	Bar intake (107 kcal/≈25 g bar): twice-daily	[206]
Elderly people, vegatarians, and vegans	Folic acid	400 μg/day	[209]
Pregnancy	Folic acid	5 mg/day	[210]
Preeclampsia	Folic acid	High-dose folic acid (4 mg/day) from 3 months before pregnancy until the entire pregnancy	[211]
CVDs and cardiovascular mortality	Genistein	-	[213]
Endothelial dysfunction and associated CVDs	Epigallocatechin-3-gallate	-	[214]
Endothelial dysfunction and associated CVDs	Curcumin	-	[215]
CVD risk	Coffee intake containing polyphenols	1–3 cups/day	[216]
Oxidative stress	Quercetin	50 mg/kg body weight daily	[108]
Oxidative stress	Melatonin and vitamin E	Melatonin: 1 mg/kg/day, Vitamin E: 125 mg/kg/day	[217]
Oxidative stress, endothelial dysfunction, and neurotoxicity	Hesperidin	100 mg/kg	[218]
AD	Intake of polyphenols	200 mL/person/day of antioxidant drink (with polyphenolic antioxidants)	[219]
CVDs	Quercetin	-	[220]
Colorectal cancer and colorectal adenoma	High folate status	-	[221]
Colorectal and esophageal cancer	Higher folate intake	Mean: 184.1–225.7 μg/day	[222]
Glaucoma	Vitamin B12 and folic acid	-	[179]
PD	Folic acid	5 mg/day	[226]
PD	Vitamin B6	Mean: 1.63 mg/day	[228]
Met adenosyltransferase I or III, SAH hydrolase, and adenosine kinase deficiency patients	Low-Met diet if plasma Met concentrations exceed 800 μmol/L	-	[230]
CBS deficiency patients	Low-Met diet and betaine	Betaine: 50 mg*/*kg/day twice (children), 3 g/day twice (adults)	[231]

## Concluding remarks and expert recommendations in the framework of 3P medicine

Hcy metabolism is crucial for regulating methionine availability, protein homeostasis, and DNA-methylation presenting; therefore, key pathways in post-genomic and epigenetic regulation mechanisms. Consequently, impaired Hcy metabolism leading to elevated concentrations of Hcy in the blood plasma is linked to the overproduction of free radicals, induced oxidative stress, mitochondrial impairments, systemic inflammation and increased risks of eye disorders, coronary artery diseases, atherosclerosis, myocardial infarction, ischemic stroke, thrombotic events, cancers development and progression, osteoporosis, neurodegenerative disorders, pregnancy complications, and delayed healing processes, among others.

**Fig. 2:**
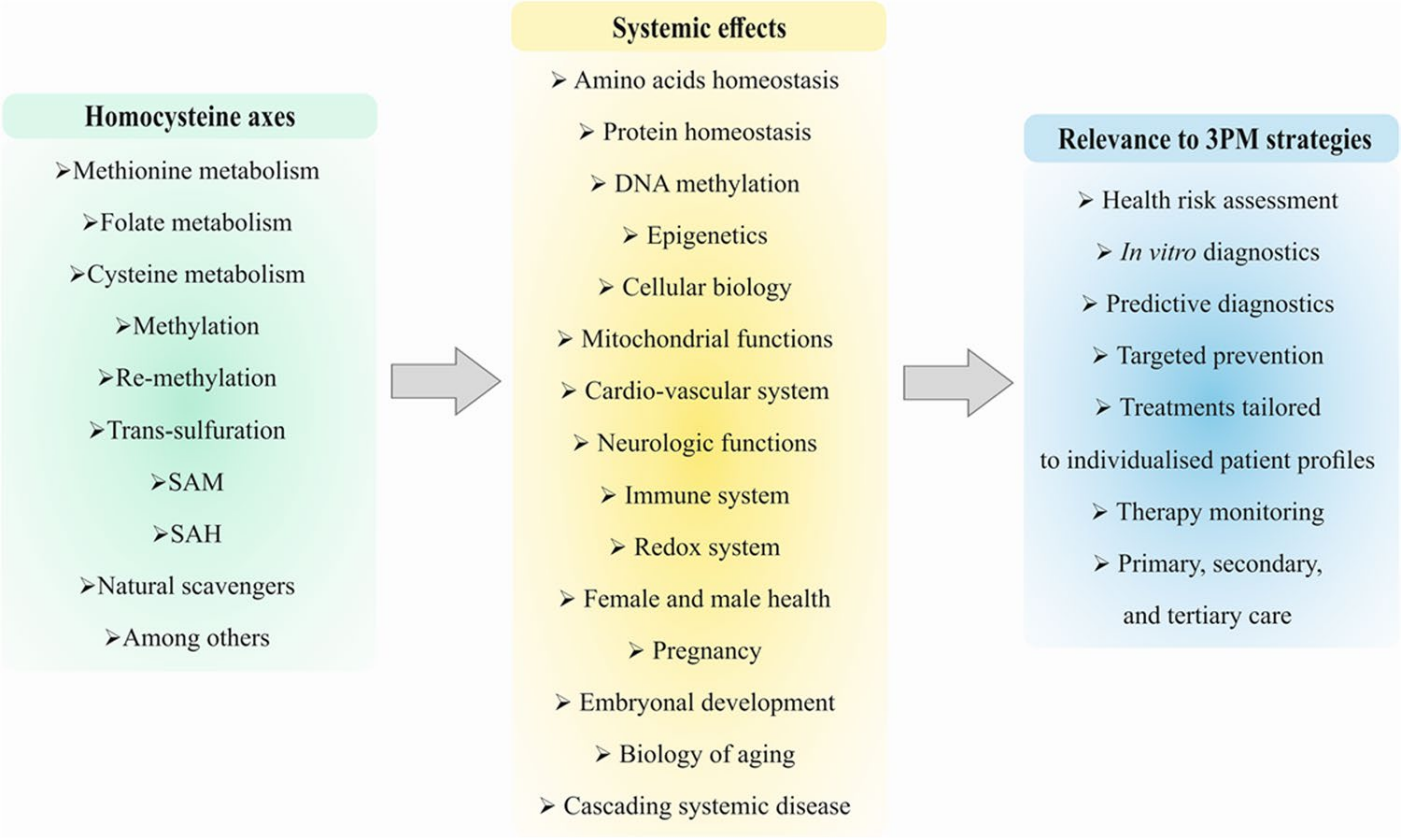
Relevance of Hcy metabolism to systemic effects and 3PM strategies; Abbreviations: SAM, S-adenosylmethionine; SAH, S-adenosylhomocysteine; 3PM, predictive, preventive, and personalized medicine

Figure 2 summarizes systemic effects by an impaired homocysteine metabolism relevant for a big number of pathological conditions. However, accurate risks assessment and individualized patient stratification are essential for the cost-effective targeted prevention and treatment algorithms tailored to the person. Innovative strategies in the framework of 3P medicine consider Hcy metabolic pathways as the specific target for in vitro diagnostics, predictive medical approaches, multi-parametric patient stratification, advanced screening programs, and preventive measures in the population as well as optimal treatments tailored to the individualized patient profiles in primary, secondary and tertiary care [63, 232–235]. To this end, in the context of severe COVID-19 complications, blood plasma Hcy was suggested as an important biomarker indicative for systemic vasculitis and inflammation, both crucial for disease outcomes prediction and targeted prevention under pandemic conditions [236]. A genetic predisposition to the altered Hcy metabolism may play a role, since there is a clear trend toward the worldwide prevalence of MTHFR 677 T and COVID-19 incidence and mortality. Statistical analysis revealed a correlation between C677 T and death from coronavirus [237].

The above highlighted facts argue in favor of innovative population screening and prevention programs involving Hcy metabolism pathways as a powerful predictive and prognostic tool as well as the cost-effective target for treatments in the framework of 3PM that is strongly recommended for updated health policy and guidelines.

## Data Availability

Not applicable.
